# Institutionalizing evaluation “of” or “by” primary care: different processes, equal importance

**DOI:** 10.1590/0102-311XEN114026

**Published:** 2026-11-09

**Authors:** Matthew Harris

**Affiliations:** 1 Imperial College London, London, U.K.

It is difficult to argue with the central premise of Felisberto et al.’s ^1^ well-written appraisal of evaluation policies in the Brazilian Unified National Health System (SUS, acronym in Portuguese). Their call for evaluation to be established as a strategic, ethical, and dialogical practice aligned with the democratic commitments of the SUS, and as a driver of social transformation that draws on concepts of planetary health, is not only timely but also well aligned with the strategic priorities of most governments waking up to the realities of a post-fossil-fuel world. Furthermore, their call to integrate different open theories, cultural perspectives, multidisciplinary knowledge and an expanded conceptualization of territory, recognition of Indigenous knowledges, and the perspectives of service users resonates completely with the Freirian perspectives that so informed the establishment of the SUS.

Felisberto et al. ^1^ open by narrating the trajectory and development of evaluation policies as the Family Health Strategy (FHS) expanded in Brazil. They describe how the establishment of the Department of Primary Care and Monitoring and Evaluation Unit, within the Brazilian Ministry of Health, signaled the importance given to evaluating primary care as it was being established. The period 2003-2006 saw further institutionalization of monitoring and evaluation through the definition of guidelines, academic partnerships, and institutional actors. The Primary Health Care Information System (SIAB, acronym in Portuguese) became publicly available, research into primary care was funded, and the development of a broad corpus of knowledge began, accelerated by the introduction of the Improvement Access and Quality National Program of Primary Health Care (PMAQ, acronym in Portuguese) payment-for-performance system and the eSUS electronic patient record system ^2^. These achievements made possible the evaluation of the more contemporary policy and political shifts, such as the introduction of the much-debated Previne Brasil - a radical reorientation of primary health care financing that some have argued undermines the core SUS principles and defunds primary care in more deprived regions ^3^, as well as the changing practices and roles of community health workers (CHW) ^4^. Felisberto et al. ^1^ argue that the re-establishment of democratic processes in 2023-2024 has led to the return to federal co-funding in line with SUS principles. Nevertheless, they argue that more needs to be done to institutionalize evaluation as the key mechanism to improve primary care services. There is an unequal response capacity of subnational entities, a tendency towards technocratization, and limited capacity to use results.

My perspectives on this are informed by having worked as a family doctor in the FHS (1999-2003), in Camaragibe (Pernambuco State) at the time that SIAB was being established. In those days, our record-keeping was completely based on pen and paper, submitted to the Municipality on a monthly basis, and used measures that bore no connection to our reality, e.g., the number of hip fractures that month. There were no opportunities to digest our data and no feedback on our performance. If the team did well, we were not rewarded. If the team did poorly, we had no way of knowing why. There was a lot of data, but no intelligence. So, in many respects the establishment of the PMAQ, eSUS, the *Brazilian National Health Survey*, and the close partnership between the Brazilian Ministry of Health, the Brazilian Institute of Geography and Statistics (IBGE, acronym in Portuguese), the Oswaldo Cruz Foundation (Fiocruz, acronym in Portuguese), and multiple academic institutions to develop policy-relevant research is a huge achievement and ought to be celebrated in Brazil. By comparison, here in the U.K., there is a significant disconnect between national policy and evaluation in primary care. We have had electronic patient records in primary care for decades, but only recently have they been linked to secondary or tertiary care services to make possible an understanding of the patient journey through the system. In many regions this is still not possible, and data is not easily available to researchers or policy makers to help understand the patterns in changing needs, health-seeking behavior, and impact of changes in services. Research into primary care services struggles to inform policy because it is not funded with that intent. The single most important issue is that we have a substantially overestimated population denominator that is wildly different from reality. Primary care registers are inflated through “ghost” patients and there are no incentives to redress this given funding is tied to practice list size. So, all in all, there is a lot to celebrate with regard to the establishment of evaluation policies in Brazil.

A key issue with Felisberto et al.’s article ^1^, however, is the conflation between Institutionalizing (capital “I”) and institutionalizing (lowercase “i”) evaluation in primary care. The former suggests creating the policies, funding, and organizations involved in the evaluation of primary care, i.e., how is it performing? The latter suggests creating a culture of evaluation within primary care, i.e., how is the community doing? These are entirely different questions and require different approaches, both of equal importance. However, evaluation of primary care is not, on its own, going to shift the onus of evaluation onto primary care teams so that they take responsibility for understanding the needs of their population and interrogating their routinely collected data to establish the impact of their day-to-day services. Establishing a culture of evaluation requires training health professionals as to the importance of it, developing both quantitative and qualitative methodological skills, access to routinely collected primary care data in easily digestible formats, and a persistent curiosity about whether locally developed interventions have worked, together with a readiness to change tactics or strategies based on the findings. This is the key difference between monitoring and evaluation. The former does not inherently require critical appraisal of the findings or establishing a program theory for change, whereas the latter does. In my day, in Camaragibe, there was a lot of monitoring, but no evaluation. And this risk remains with the current institutional arrangements around Previne Brasil, which evolved from the PMAQ - a lot of data, but perhaps no intelligence ^5^. If it is not already, the Residency in Family Health ought to include methodological training for robust evaluations in primary care, and the technical qualification for CHW ^6^ ought to include training in participatory evaluation and action research.

But where Institutionalizing evaluation (capital “I”) is important is in answering some of the big questions in primary care that individual units, or even municipalities, cannot address. For example, despite Brazil having the world’s largest primary care system based on CHWs, there has never been, to my knowledge, a cluster randomized controlled study to explore the impact of this important cadre on health outcomes and health-seeking behavior in Brazil. Multiple cross-sectional studies at national level have been conducted but these either fall afoul of the ecological fallacy or do not adequately distinguish between the FHS unit, i.e., the wider team of doctor, nurse, nurse auxiliary, and CHWs, and the impact of the CHWs exclusively as an important cadre. In a recent systematic review (soon to be published), our team found dozens of studies in Brazil on the impact of CHWs in different clinical domains, but none were randomized or controlled. Considering the debates around CHWs in Brazil and internationally - a cadre that is still undervalued and unappreciated in part because of a lack of robustly controlled evidence - Brazil is still in an enviable position to conduct such studies at scale due to the close institutional arrangements between the Brazilian Ministry of Health and renowned research entities such as Fiocruz. It shouldn’t miss this opportunity.

## Data Availability

Not applicable.
