# Supplementary material for: Institutionalizing evaluation “of” or “by” primary care: different processes, equal importance
Source: Cad Saude Publica. 2026 Nov 9;42(Suppl 2):e00114026. doi: 10.1590/0102-311XEN114026 (PMC13750636; doi:10.1590/0102-311XEN114026)
Supplement: Translation

# Translation: Institucionalização da avaliação “da” ou “pela” atenção primária à saúde: processos diferentes, igual importância

**DOI:** 10.1590/0102-311XPT114026

**Authors:** Matthew Harris

**Affiliations:** 1 Imperial College London, London, U.K.

É difícil contestar a premissa central da bem fundamentada análise de Felisberto et al. ^1^ sobre as políticas de avaliação no Sistema Único de Saúde (SUS). O apelo dos autores para que a avaliação seja estabelecida como uma prática estratégica, ética e dialógica, alinhada aos compromissos democráticos do SUS e concebida como um agente de transformação social fundamentado em conceitos de saúde planetária, não é apenas oportuno, mas também está em consonância com as prioridades estratégicas da maioria dos governos que despertam para as realidades de um mundo pós-combustíveis fósseis. Além disso, a defesa da integração de diferentes teorias abertas, perspectivas culturais, conhecimentos multidisciplinares, uma conceituação ampliada de território, reconhecimento dos saberes indígenas e as perspectivas dos usuários dos serviços de saúde dialoga plenamente com as perspectivas freirianas que tanto influenciaram a constituição do SUS.

Felisberto et al. ^1^ iniciam seu trabalho narrando a trajetória e o desenvolvimento das políticas de avaliação à medida que a Estratégia Saúde da Família (ESF) se expandiu no Brasil. Os autores descrevem como a criação do Departamento de Atenção Básica e a Coordenação de Monitoramento e Avaliação, no âmbito do Ministério da Saúde, sinalizaram a importância atribuída à avaliação da atenção primária à saúde durante seu processo de consolidação. O período de 2003 a 2006 foi marcado por uma maior institucionalização do monitoramento e da avaliação por meio da definição de diretrizes, parcerias acadêmicas e atores institucionais. O Sistema de Informação da Atenção Básica (SIAB) tornou-se publicamente acessível, pesquisas sobre atenção primária à saúde passaram a receber financiamento e iniciou-se o desenvolvimento de um amplo corpo de conhecimentos, impulsionado pela introdução do Programa de Melhoria do Acesso e da Qualidade da Atenção Básica (PMAQ), um sistema de pagamento por desempenho, e do sistema prontuário eletrônico eSUS ^2^. Esses avanços possibilitaram a avaliação das mudanças políticas e programáticas mais contemporâneas, como a implementação do amplamente debatido Previne Brasil - uma reorientação radical do financiamento da atenção primária à saúde que, segundo alguns autores, enfraquece os princípios centrais da SUS e reduz o financiamento da atenção primária em regiões mais vulneráveis ^3^, bem como das mudanças nas práticas e nos papéis dos agentes comunitários de saúde (ACS) ^4^. Felisberto et al. ^1^ argumentam que o restabelecimento dos processos democráticos, em 2023-2024, levou ao retorno do cofinanciamento federal em conformidade com os princípios do SUS. Ainda sim, os autores defendem que mais esforços são necessários para institucionalizar a avaliação como o principal mecanismo para aprimorar os serviços de atenção primária. Persistem desigualdades na capacidade de resposta das entidades subnacionais, uma tendência à tecnocratização e limitações na capacidade de utilizar resultados das avaliações.

Minhas perspectivas sobre essa questão são informadas pela minha experiência como médico de família no ESF (1999-2003), em Camaragibe (Estado de Pernambuco), no período em que o SIAB estava sendo implementado. Naquela época, nossos registros eram feitos integralmente em papel, enviados mensalmente ao município e baseados em indicadores que não tinham qualquer relação com nossa realidade, como, por exemplo, o número de fraturas de quadril registradas naquele mês. Não havia oportunidades de analisar dados produzidos nem de receber devolutivas sobre nosso desempenho. Se a equipe apresentasse bons resultados, não havia reconhecimento. Se a equipe tivesse desempenho insatisfatório, não tínhamos meios de compreender os motivos. Havia uma grande quantidade de dados, mas não havia inteligência. Assim, em muitos aspectos, a criação do PMAQ, do eSUS, da *Pesquisa Nacional de Saúde* e a estreita parceria entre o Ministério da Saúde, o Instituto Brasileiro de Geografia e Estatística (IBGE), a Fundação Oswaldo Cruz (Fiocruz) e várias instituições acadêmicas para desenvolver pesquisas relevantes para a formulação de políticas públicas representam uma grande conquista e devem ser celebradas no Brasil. Em comparação, aqui no Reino Unido, há uma desconexão significativa entre políticas nacionais e processos de avaliação na atenção primária à saúde. Temos prontuários eletrônicos de pacientes na atenção primária há décadas, mas apenas recentemente esses registros passaram a ser integrados aos serviços de atenção secundária e terciária, tornando possível compreender a trajetória do paciente ao longo do sistema de saúde. Em muitas regiões, essa integração ainda não é possível, e os dados não estão facilmente disponíveis para pesquisadores ou formuladores de políticas públicas, dificultando a compreensão dos padrões de mudança nas necessidades de saúde, dos comportamentos de busca por cuidados e do impacto das mudanças nos serviços. Pesquisas sobre serviços de atenção primária enfrentam dificuldades para informar a formulação de políticas, pois não são financiadas com esse propósito. A questão mais importante é que temos um denominador populacional substancialmente superestimado, muito distante da realidade. Os registros de atenção primária encontram-se inflados pela presença de pacientes “fantasmas”, e não há incentivos para corrigir essa situação, uma vez que o financiamento está vinculado ao tamanho da lista de pacientes de cada unidade. Portanto, de modo geral, há muito a celebrar em relação ao estabelecimento de políticas de avaliação no Brasil.

Entretanto, uma questão central do artigo de Felisberto et al. ^1^ é a confusão entre Institucionalizar (com “I” maiúsculo) e institucionalizar (com “i” minúsculo) a avaliação na atenção primária. O primeiro conceito refere-se à criação de políticas, mecanismos de financiamento e organizações voltados à avaliação da atenção primária, isto é, à pergunta: Como ela está funcionando? O segundo refere-se à construção de uma cultura de avaliação dentro da própria atenção primária, isto é, à pergunta: Como a comunidade está se saindo? Essas questões são totalmente diferentes e exigem abordagens distintas, embora ambas tenham igual importância. Contudo, avaliação da atenção primária, por si só, não será suficiente para transferir a responsabilidade pela avaliação às equipes de atenção primária, de modo que elas assumam o compromisso de entender as necessidades da população sob sua responsabilidade e de analisar os dados coletados rotineiramente, com o objetivo de estabelecer o impacto de seus serviços cotidianos. Estabelecer uma cultura de avaliação exige a formação de profissionais de saúde quanto à sua importância, o desenvolvimento de habilidades metodológicas quantitativas e qualitativas, o acesso aos dados rotineiramente coletados atenção primária em formatos facilmente interpretáveis e uma curiosidade persistente em relação à eficácia das intervenções desenvolvidas localmente, juntamente com a disposição para modificar táticas ou estratégias com base nos resultados obtidos. Essa é a principal diferença entre monitoramento e avaliação. O primeiro não exige, necessariamente, uma análise crítica das descobertas nem o estabelecimento de uma teoria de programa para a mudança, enquanto o segundo exige. Na época em que trabalhei em Camaragibe, havia muito monitoramento, mas nenhuma avaliação. E esse risco permanece nos atuais arranjos institucionais relacionados ao Previne Brasil, que evoluiu a partir do PMAQ: muitos dados, mas talvez nenhuma inteligência ^5^. Caso ainda não contemple, a Residência em Saúde da Família deveria incluir formação metodológica para avaliações rigorosas na atenção primária, e a qualificação técnica dos ACS ^6^ deveria incluir capacitação em avaliação participativa e pesquisa-ação.

Entretanto, é na Institucionalização da avaliação institucionalizada (com “I” maiúsculo) que reside sua importância para responder a algumas das grandes questões da atenção primária que unidades individuais, ou mesmo municípios, não são capazes abordar. Por exemplo, apesar de o Brasil possuir o maior sistema de atenção primária do mundo baseado em ACS, nunca houve, até onde sei, um estudo randomizado controlado por conglomerados para explorar o impacto dessa importante categoria profissional sobre os desfechos em saúde e os comportamentos de busca por cuidados no Brasil. Diversos estudos transversais em nível nacional foram realizados, mas estes ou incorrem na falácia ecológica ou não conseguem distinguir adequadamente o impacto do ESF - isto é, da equipe mais ampla composta por médicos, enfermeiros, auxiliares de enfermagem e ACS - do impacto específico dos ACS enquanto categoria profissional essencial. Em uma revisão sistemática recente (ainda a ser publicada), nossa equipe encontrou dezenas de estudos realizados no Brasil sobre o impacto dos ACS em diferentes domínios clínicos, mas nenhum foi randomizado ou controlado. Considerando os debates em torno os ACS no Brasil e internacionalmente - uma categoria profissional ainda subvalorizada e pouco reconhecida, em parte devido à falta de evidências robustas provenientes de estudos controlados - o Brasil ainda ocupa uma posição privilegiada para conduzir tais estudos em larga escala, em razão dos estreitos arranjos institucionais entre o Ministério da Saúde e instituições de pesquisa renomadas, como a Fiocruz. O país não deveria perder essa oportunidade.

## Data Availability

Não se aplica.
